# Growth Hormone Response to L-Arginine Alone and Combined with Different Doses of Growth Hormone-Releasing Hormone: A Systematic Review and Meta-Analysis

**DOI:** 10.1155/2022/8739289

**Published:** 2022-11-23

**Authors:** Parvin Goli, Maryam Yazdi, Motahar Heidari-Beni, Roya Kelishadi

**Affiliations:** ^1^Department of Pediatrics, Child Growth and Development Research Center, Research Institute for Primordial Prevention of Non-Communicable Disease, Isfahan University of Medical Sciences, Isfahan, Iran; ^2^Department of Nutrition, Child Growth and Development Research Center, Research Institute for Primordial Prevention of Non-Communicable Disease, Isfahan University of Medical Sciences, Isfahan, Iran

## Abstract

**Background:**

Arginine (ARG) can modulate growth hormone (GH) release by suppressing its endogenous inhibitory regulator, somatostatin. ARG also induces the release of the GH-releasing hormone (GHRH). This study aims to review the effects of L-arginine supplementation alone and combined with GHRH in different doses on GH secretion.

**Methods:**

In this systematic review and meta-analysis, an electronic literature search was conducted on Medline database (PubMed), Scopus, and Web of Science databases. All eligible studies were randomized clinical trials that reported the effects of ARG supplementation alone or with GHRH on GH levels. Mean difference (MD) and 95% confidence intervals (CI) were computed as the effect size.

**Results:**

Meta-analyses showed significant effects of ARG alone on GH release (MD = 10.07, 95% CI: 7.87, 12.28). Moreover, the response of GH was greater with ARG in combination with GHRH (MD = 24.96, 95% CI: 17.51, 32.42). There was no significant difference between the patients and healthy individuals and between oral and injection use of ARG. The systematic review revealed the important role of ARG in combination with other amino acids on GH secretion in patients with GH deficiency.

**Conclusion:**

This study revealed that in GH-deficient individuals, high doses of ARG supplementation in combination with GHRH and/or other amino acids might have potential therapeutic effects on increasing GH concentrations. These findings propose that ARG supplementation can be considered as a potential stimulator in management of GH deficiency.

## 1. Introduction

Growth hormone (GH) is a metabolic hormone which stimulates lipolysis and influences catecholamine and insulin functions. It maintains blood glucose levels by reducing carbohydrate metabolism. GH also plays a role in protein synthesis through the release of insulin-like growth factor (IGF-I) [1]. Polymorphisms of some genes such as IL-12p40 are associated with concentration levels of GH and IGF-1 [2]. GH release in a pulsatile pattern is controlled by the hypothalamus, which affects the anterior pituitary gland by GH-releasing hormone (GHRH) as a stimulator and somatostatin as an inhibitor [3, 4]. The peaks occur at night and are associated with slow wave sleep [5]. Higher levels of GH cause auto-negative feedback in GH secretion. There are numerous physiological stimulators for GH secretion, including gender, body composition, nutrition, sleep, fitness level, and sex steroid hormones [6].

Growth hormone deficiency (GHD) can occur in hypothalamic or pituitary diseases that may lead to disturbance in lipoproteins, impairment in carbohydrate metabolism, decreases in lean body mass, increases in body fat, and short stature or impaired growth velocity in the pediatric age group [7, 8]. GH therapy is used in hypopituitaric patients with severe GHD and leads to increase in lean body mass and muscle strength within months of treatment initiation [9]. Resting GH secretion could potentially be stimulated by the intravenous administration of various amino acids such as arginine (ARG), methionine, phenylalanine, lysine, or histidine [10, 11]. Some studies have shown the effectiveness of ARG infusion or oral ingestion of ARG on GH release in both men and women [12]. Moreover, intravenous ARG infusion has been used clinically to diagnose the responsiveness of the GH axis in GHD [13]. Some studies have indicated that the combination of ARG with some amino acids such as glutamine or lysine or some hormones such as GHRH has led to higher GH secretion [14, 15]. Recent animal study showed that ARG supplementation increased significantly the levels of GHRH, GH and IGF-1 [16]. A study on children showed that intervention with different doses of GH did not influence of height significantly [17]. However, there are inconsistent findings and some previous studies did not confirm the effect of ARG on increasing GH secretion [18]. Different doses of ARG in studies have also led to discrepancies between the results. Therefore, a comprehensive review and summary of the literature are needed.

Clinically, GHD must be shown biochemically by single provocative testing [19]. According to the findings, GHRH alone was not a reliable provocative test [19, 20]; the diagnostic value of this neurohormone can be increased in combination with substances with GH-releasing activity, such as ARG [21–23].

Studies have shown different findings related to GH response to L-arginine supplementation alone and in combination with GHRH in different doses. Therefore, this systematic review and meta-analysis aims to provide a summary of the literature evaluating the effects of L-arginine supplementation alone and along with different doses of GHRH on GH secretion.

## 2. Methods

This systematic review protocol was registered in the International Prospective Register of Systematic Reviews, based on the protocol of Preferred Reporting Items for Systematic Reviews and Meta-Analysis (PRISMA 2020) [24] and was registered on PROSPERO (Registration number: CRD42021242954) (Appendix 1, Supplementary section).

### 2.1. Literature Search and Selection

**Figure 1:**
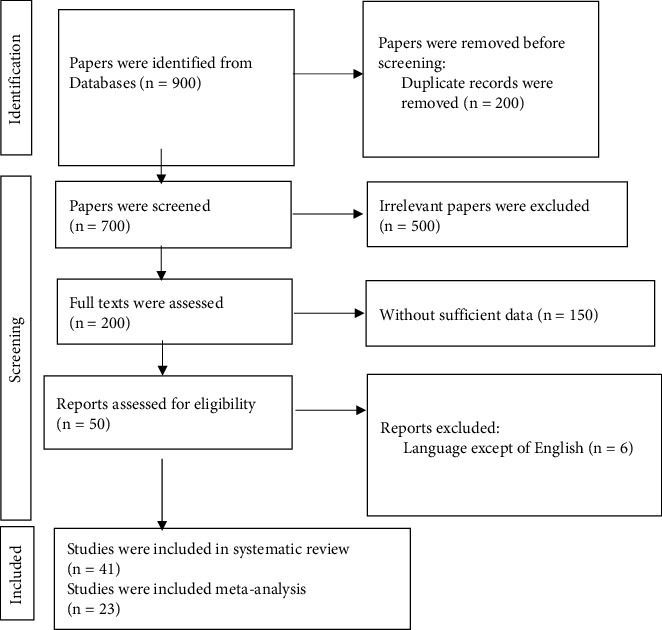
Flow diagram of the study selection process for this systematic review and meta-analysis.

Relevant articles were searched in the Medline (PubMed), Web of Science, and Scopus databases. Databases were searched up to end of January, 2021, and updated till October, 2022. Medical subject heading (MeSHs) was used in the search strategy. The following terms were logically combined to search sources related to the main topic of the analysis without date restrictions: (((“growth hormone” OR “human growth hormone” OR “GH”) AND (“Arginine” OR “L-arginine” OR “LArg” OR “Arg”) AND (“Intervention study” OR “controlled trial” OR “randomized” OR “randomized controlled trial” OR “randomized clinical trial” OR RCT))) (Appendix 2, Supplementary section). Electronic literature searches were complemented with reference list and citation searches. The references of included studies and reviews were checked by backward and forward snowball searches. The research process was conducted by two authors (PG and MHB) independently. Study selection was also done independently, duplications were removed, and after titles and abstracts were screened, full-text articles of eligible studies meeting the inclusion criteria were assessed (Figure 1). Any disagreements in this regard were resolved through discussion with the third researcher (RK). A flow diagram for the study selection process is shown in Figure 1.

### 2.2. Eligibility Criteria

Only English language articles and human studies were included. All human randomized clinical trials (RCTs), either parallel or crossover designs, which reported the effects of ARG supplementation alone and/or with GHRH on GH levels were selected. Animal studies, in vitro, and in vivo studies, studies that combined L-arginine with other peptides or hormones like arginine + insulin or lysine, and studies reporting the effects of ARG in pregnancy or postpartum were excluded.

### 2.3. Data Extraction

The following information was extracted from the full text of included studies: first author, publication year, location of the study, total sample size, type and dose of intervention and placebo, study design, study duration, outcomes and results. In cases where relevant data was lacking, the corresponding authors were contacted by e-mail for help. Two investigators (PG and MHB) extracted the data separately to minimize potential errors. Any disagreement was resolved by the third reviewer (RK).

### 2.4. Quality Assessment of Studies

Cochrane's collaboration tools were used to assess the quality of studies [25]. Two researchers (PG and MHB) independently evaluated the methods and quality of eligible studies using Cochrane's collaboration tools, which includes seven domains: (1) random sequence generation; (2) allocation concealment; (3) blinding of participants and personnel; (4) blinding of outcome assessment; (5) incomplete outcome data; (6) selective reporting; and (7) other sources of bias [25]. Each scope was further classified into three classes: low risk, high risk, and unclear risk of bias. According to the guidelines, the general quality of each study was considered as good (low risk for more than two cases), fair (low risk for two cases), or weak (low risk for fewer than two cases) [25].

The GRADE framework rated the strength of the evidence for outcome (GH secretion) as moderate (Supplementary Table 1).

### 2.5. Statistical Analysis

Intervention effects were determined using mean differences (MDs), calculated by peak values of posttreatment and pretreatment GH. Data from intent-to-treat (ITT) analyses were preferred over data from modified-ITT or per protocol analyses. Individual study weights were calculated as the inverse of the variance. Weighted averages and 95% confidence intervals were pooled using a random effects model. The *I*^2^ statistic (percentage of the total variation across studies due to heterogeneity) was calculated to determine the between-study statistical heterogeneity. A meta-regression analysis was conducted using the metareg command in STATA to assess the relationship between received dosages of ARG and MDs. A sensitivity analysis was conducted by leaving one study each time. Publication bias was assessed with funnel plot, Beg's, and Egger's tests.

We used Kappa statistics to assess interrater agreement between reviewers for study inclusion and assessment of the risk of bias [26]. Values of kappa between 0.40 and 0.59 have been considered to reflect fair agreement, between 0.60 and 0.74 to reflect good agreement, and 0.75 or more to reflect excellent agreement [27]. Statistical analyses were performed using STATA version 14 (StataCorp LP, College Station, TX, USA) software. Two-tailed significant probability was considered less than 0.05.

## 3. Results

### 3.1. Study Selection

After the eligibility criteria were screened, 41 eligible studies were included in the systematic review and 23 articles in the meta-analysis [6, 8, 13, 28–65]. Interrater agreement between reviewers for study selection was excellent (Kappa statistics = 0.89). A flow diagram for the study selection process is shown in Figure 1.

### 3.2. Study Characteristics

All studies except two [28, 65] measured the GH in plasma. Most studies used infusion type supplements; however, four studies assessed the effect of oral supplements [6, 28, 33, 34, 36]. Twenty-one studies assessed the response of GH to ARG alone [6, 8, 11–13, 28–30,32, 34, 36, 38, 44, 50, 53–55, 62, 63, 65, 66], and twenty-one studies investigated the response of GH to ARG + GHRH [6, 8, 31, 33, 35, 37, 40–43, 47–49, 52, 56–59, 61, 67, 68]. Some studies demonstrated the responsiveness of GH to combinations of ARG with other amino acids like lysine and ornithine [30, 39]. Supplementary Table 2 provides an overview of the study characteristics and outcome measures.

### 3.3. Study Quality

Overall, 42 studies reported low risk on randomization and 22 studies reported low risk on blinding. Following the results of the Cochran Risk of Bias tool, an average of 3 studies in every part had an overall unclear risk of bias. Supplementary Table 3 shows the risk of bias assessment of the included studies. Agreement of the risk of bias assessment was excellent (Kappa statistics = 0.82).

### 3.4. Effects of ARG on Growth Hormone Levels

**Figure 2:**
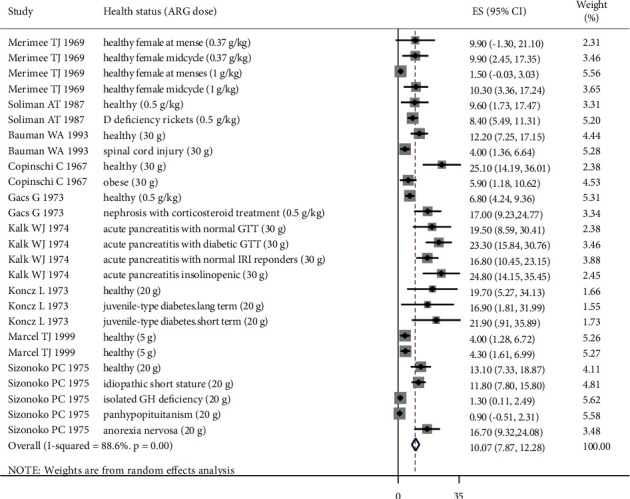
Forest plot of the effects of arginine (ARG) on the growth hormone (GH) level. The individual effect sizes are identified as mean difference (MD) with lower and upper limits (95% CIs) for each study. The overall summary effect sizes of the meta-analysis are noted as a diamond.

The findings of the meta-analysis on 30 extracted sets of data showed that ARG was associated with a significant increase in GH level (MD = 10.07 *μ*g/ml, 95% CI: 7.87, 12.28). There was evidence of heterogeneity between the included studies (*I*^2^ = 88.6%, *p* < 0001) (Figure 2).

### 3.5. Subgroup Analysis and Meta-Regression

Subgroup analyses were performed to describe potential sources of heterogeneity. Oral ARG administration showed a smaller increase in GH levels in comparison with ARG injection (*p* = 0.141) (Table 1). The effects of ARG on GH levels were not different between adults and children (*p* = 0.649) or between healthy and non-healthy subjects (*p* = 0.390).

**Figure 3:**
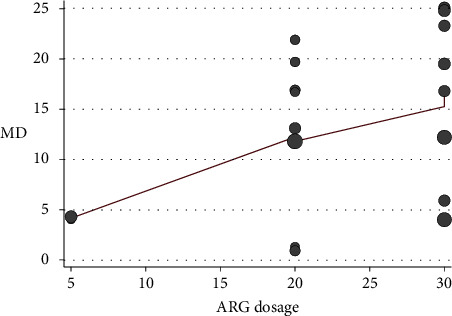
Scatter plot of ARG dosage vs. mean difference of posttreatment GH level from baseline. The smoothed line was fitted using the LOWESS method.

The association of study-specific MDs of GH with ARG dosage was assessed using meta-regression. There was a marginally significant association between a higher ARG dose and GH secretion (adjusted *R*^2^ = 17.50%, *p* = 0.061) (Table 1, Figure 3).

### 3.6. Effects of ARG + GHRH on Growth Hormone Levels

**Figure 4:**
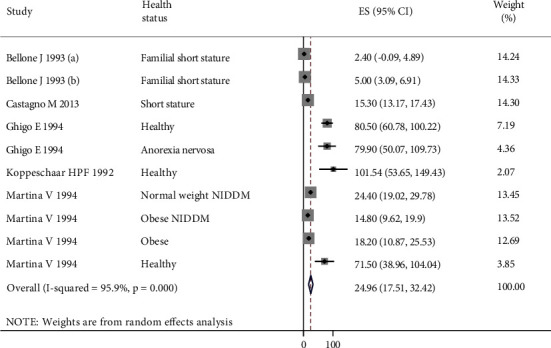
Forest plot of the effects of ARG + GHRH on the GH level. The individual effect sizes are identified as mean difference (MD) with lower and upper limits (95% CIs) for each study. The overall summary effect sizes of the meta-analysis are noted as a diamond.

The mean differences of GH level after treatment with a combination of ARG and GHRH from baseline value are shown in Figure 4. The findings of meta-analyses on 10 extracted sets of data showed that ARG + GRHG increased GH levels up to 24.96 *μ*g/ml (95% CI: 17.51, 32.42), which is higher than seen in treatments with ARG alone.

### 3.7. Publication Bias

**Figure 5:**
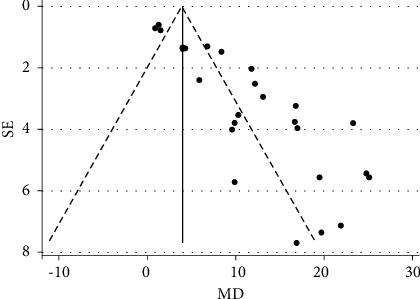
Funnel plot of MDs of the GH level of post- and pre-treatment with ARG.

There was evidence of publication bias regarding the effects of ARG on GH levels according to Beg's test (*p* = 0.028) and Egger' test (*p*_slop_ = 0.123, *p*_bias_ < 0.001). The funnel plot of included studies was also asymmetric (Figure 5). There was also evidence of publication bias regarding the effect of ARG + GHRH on GH levels according to Egger' test (*p*_slop_ = 0.461, *p*_bias_ = 0.013).

## 4. Discussion

To the best of the authors' knowledge, this is the first meta-analysis to assess the effects of ARG alone and ARG combined with different doses of GHRH on GH concentrations in GH deficient patients. Despite the heterogeneity in some parts, this study indicated a progressively larger GH response with increasing ARG doses and the effectiveness increased when ARG was combined by other factors, like lysine or GHRH.

ARG affects GH secretion by regulating hypothalamic-pituitary axis [69]. Currently, ARG is one of the classic provocative tests for the diagnosis of GHD [70]. It has been previously reported that a slight increase in plasma GH concentration occurs due to ARG infusion in patients with genetically determined monotropic GHD [13].

The present systematic review indicated a higher response to ARG during midcycle in comparison to active menstrual period. This may be explained by the greater estrogen secretion in midcycle. A previous study showed that estrogen pretreatment supplement boosted the GH response to ARG [71].

The current study found that the GH response to ARG was similar in both children and adults, but previous studies showed the increasing response of GH secretion to ARG in elderly individuals [72, 73]. The probable mechanism is that ARG suppresses the endogenous somatostatin release, which decreases the age-related effect of hexarelin on GH secretion in elderly subjects. Based on both animal and human studies, human somatostatinergic hyperactivity in old ages has been shown to confirm the decline in GH stimulation as occurring along with the failure effect of ARG on the GH response to hexarelin [59, 74, 75].

Noticeable age-related hypothalamic somatostatinergic activity increased hexarelin's antagonism of somatostatin at the pituitary level [31]. On the other hand, a decrease in muscle mass occurs during human aging, which is in accordance with a decrease in GH concentration. Moreover, enhanced muscle mass is associated with GH release. Therefore, supplements like ARG may be beneficial for increasing GH levels in elderly subjects [32]. ARG supplements may increase IGF-I in plasma as well as muscle mass. Thus, athletes may use supplements to increase performance by stimulating GH [8]. According to the current results, GH levels could be administered by oral ARG, but the timing of the usage might be important. Oral ingestion has a slower process than intravenous administration, so a delayed response should be expected. The first rise in GH concentration was seen approximately 30 min after ARG ingestion. The peak level of GH then occurred within 30–60 min after the onset of the initial rise. This suggests that when combined with exercise, the exercise session should commence approximately 30 min after ARG ingestion. Although ARG also increases GH levels at rest, exercise after ARG intake increases GH levels even higher [32].

As a result of increasing GH, ARG may affect height by the assessment of growth velocity in normal children that take ARG, indicated the faster increase in height of 0.33 cm/year compared with normal children who have not used ARG. However, this association depended on dose response, which was significant in doses of more than 2.2 g/d [64]. Animal studies have shown dose-dependent increases in body weight due to ARG supplementation [64].

It cannot be concluded that ARG supplementation can or cannot be considered a clinical nutritional strategy for the prevention or treatment of short stature [64]. Based on the evidence, no change in growth velocity was seen in children with an idiopathic short stature [50].

According to the current review, oral ARG provoked a significant greater GH response with higher doses. Increasing ARG concentration can be transported to the hypothalamus and easily affect the GH stimulator. The pituitary can control degrees of GH secretion by suppressing somatostatin concentrations and by releasing more GH with a greater ARG signal [33]. The results of previous studies showed a 72% increase in resting GH level when 9 g of oral ARG was administered daily over a four-week period and a 6 *μ*g/L increase in GH levels when 1.2 g of oral ARG was administered [54].

The current data assessment showed a similar dose-dependent effect of the injectable and ingestible forms of ARG on GH. Previous studies have also reported that a dose of more than 1/12 g per pound of body weight resulted in a significant increase in GH in men, while doses lower than 1/12 per pound of body weight achieved significant results in women [65]. A study on men showed an increase in GH level from 2.4 to 32 *μ*g/L when 30 g of ARG was infused over 30 min [68]. The present systematic review indicates a higher response to ARG during midcycle in comparison to an active menstrual period. This may be explained by the greater estrogen secretion in midcycle. A previous study showed that estrogen pretreatment supplement boosted the GH response to ARG [71].

A comparison of the data showed that the time the peak of GH was reached was not affected by different doses of ARG. While IV infusion of ARG is a faster method of entering ARG into the plasma, oral ARG also increased GH secretion only about 10 min slower.

Furthermore, there was evidence of improvement in the GH response to ARG when combined with other amino acids [54]. A combination of ARG and lysine led to a 5-fold increase in GH compared with ARG alone [6]. ARG and pyridostigmine improved the GH response to GHRH and stimulated the somatostatin-mediated negative GH auto-feedback mechanism [72]. The combination of ARG with amino acids may induce more absorption of the supplements, and thus, it could be more easily transported to the hypothalamus.

The present study showed that GH levels increased more with ARG + GHRH than with ARG or GHRH alone. ARG counteracted the maximal pituitary somatotroph responsiveness to GHRH and suppressed the endogenous somatostatin release, so the GH release on the hypothalamic level increased [67]. On the other hand, somatostatin release in the hypothalamic fluctuates spontaneously; thus, GHRH alone was unable to reliably explore the secretory capacity of somatotroph cells and had no diagnostic reliability. In addition, there was variability in the somatotroph responsiveness to GHRH alone [19].

The exact mechanisms of inducing GH release by ARG was not clear, but it was clarified that ARG acts as a neurotransmitter at the central level [72]. The evidence indicates that ARG potentiated the GH response to GHRH; hence, GHRH acted as a powerful and reproducible stimulus of somatotroph secretion as ARG has somatostatin-mediated action [58]. This evidence resulted in the hypothesis that ARG inhibited hypothalamic somatostatin [19]. Some evidence supports the hypothesis that ARG has somatostatin-mediated action [35]. ARG affects GH release by resisting the inhibitory effect of glucose and free fatty acids [3, 5]. These inhibitors act through enhanced hypothalamic somatostatin release [19, 68]. Herein, ARG counteracted the negative feedback action exerted by GH [67]. This evidence implies that testing with GHRH in combination with ARG represents a very powerful and reproducible stimulus of GH secretion. On the other hand, somatostatin release in the hypothalamic fluctuates spontaneously; thus, GHRH alone is unable to reliably explore the secretory capacity of somatotroph cells and has no diagnostic reliability as there is variability in the somatotroph responsiveness to GHRH alone [19].

There are some factors that they might be associated with GH secretion. Studies showed that heat exposure, physical activity, age, gender, body composition, muscle mass, visceral fat, central adiposity, some hormones, blood glucose, and insulin levels influence GH secretion [76, 77]. However, the association between body fat, glucose disposal, and GH secretion remains unclear. The inclusion of nonresponders in the findings can lead to inconsistency between the studies in the GH responses reported [65, 78].

Findings showed that low GH has influence on intramyocellular lipids (IMCL) and intrahepatic lipid accumulation and it may be associated with insulin resistance in obesity. Intrahepatic lipid concentration can be considered as a determinant of peak GH levels. So, it can be concluded that fat depots other than visceral fat may decrease GH secretion. However, interventional studies are needed for more investigation of this hypothesis. In addition, IMCL of tibialis anterior muscle is a significant determinant of peak GH levels [79, 80].

Studies have shown that the reason for the gender difference in GH secretion is due to estrogen. There are greater GH responses during the menstrual cycle and the time of increased estrogen production. Elevated ARG-induced GH secretion was showed in men after short-term exposure to estrogen [81].

Exercise might affect GH secretion by inhibition of hypothalamic somatostatin release and stimulation of GHRH or ghrelin release [82]. To provide preference for the combination of GHRH with ARG, it has also been shown that the GH response to this stimulus was refractory to the negative feedback action of IGF-1 [5] and, above all, it was basically independent of aging [11]. The GH response to GHRH + ARG in elderly subjects has been found preserved and similar to that recorded in young adults [59] and normally growing children [73]. The rational basis for considering GHRH + ARG as a reliable provocative test of somatotroph function is its potency, reproducibility, and refractoriness to the majority of negative influences on GH secretion.

## 5. Conclusion

This meta-analysis revealed the potential effect of ARG in combination with GHRH on GH secretion of GH deficient patients. ARG in high doses potentially stimulates GH responsiveness in these patients. Moreover, the effect of ARG was increased when used along with other amino acids. Therefore, ARG supplementation could be considered as a potential stimulator treatment for both adults and children with GH deficiency.

## Figures and Tables

**Table 1:** Subgroup analysis for the effect of arginine on growth hormones.

		MD (95% CI)	P	*I * ^2^%	P_meta−regression_
*Overall injection*		10.08 (7.87,12.28)	<0.001	88.6	
	IV	10.95 (8.49,13.42)	<0.001	89.5	0.141
	Oral	4.15 (2.24,6.07)	0.878	0.0	

*ARG type*	Hydrochloride	8.57 (5.61,11.52)	0.005	70.2	0.217
	Mono-hydrochloride	13.91 (9.86,17.96)	<0.001	92.0	

*Region*	America	8.11 (5.31,10.92)	<0.001	78.6	0.338
	Africa	16.35 (10.16,22.54)	<0.001	79.7	0.519
	Europe	8.41 (4.68,12.14)	<0.001	92.5	

*Dose*	0.37 g/kg	9.9 (3.7,16.1)	1	0.0	0.061
	0.5 g/kg	8.97 (5.87,12.07)	0.102	51.6	
	1 g/kg	5.22 (-3.3,13.75)	0.015	83.0	
	5–10 g	4.15 (2.24,6.07)	0.878	0.0	
	20 g	9.76 (5.61,13.91)	<0.001	90.4	
	30 g	15.47 (9.42,21.53)	0	87.8	

*Age group*	Adult	10.99 (7.69,14.29)	<0.001	87.0	0.649
	Children	9.38 (6.07,12.69)	<0.001	90.6	

*Health status*	Healthy	8.65 (5.7,11.59)	<0.001	81.9	0.39
	Nonhealthy	11.58 (8.15,15)	<0.001	91.7	

MD: mean difference, CI: confidence interval.

## Data Availability

The obtained data would be disclosed upon reasonable request.

## Supplementary Materials

Supplementary Materials Supplementary Table 1: GRADE evidence profile: Effect of Vitamin ARG and ARG + GRGH on GH. Supplementary Table 2: Study characteristics. Supplementary Table 3: Risk of bias assessment of the studies included in this meta-analysis. Appendix 1: PRISMA 2020 Checklist. Appendix 2: Search terms for Medline (PubMed).
