# Supplementary material for: Growth Hormone Response to L-Arginine Alone and Combined with Different Doses of Growth Hormone-Releasing Hormone: A Systematic Review and Meta-Analysis
Source: Int J Endocrinol. 2022 Nov 23;2022:8739289. doi: 10.1155/2022/8739289 (PMC9712012; doi:10.1155/2022/8739289)
Supplement: Supplementary Materials — Supplementary Table 1: GRADE evidence profile: Effect of Vitamin ARG and ARG + GRGH on GH. Supplementary Table 2: Study characteristics. Supplementary Table 3: Risk of bias assessment of the studies included in this meta-analysis. Appendix 1: PRISMA 2020 Checklist. Appendix 2: Search terms for Medline (PubMed). [file 8739289.f1.zip › Appendix 2 (1).docx]

Search terms for PubMed

(("Arginine"[Title/Abstract] OR "L-arginine"[Title/Abstract] OR "LArg"[Title/Abstract] OR "Arg"[Title/Abstract] OR "arginine alpha-ketoglutarate"[Title/Abstract] OR "AAKG"[Title/Abstract]) AND ("human growth hormone"[Title/Abstract] OR "HGH"[Title/Abstract] OR "GH"[Title/Abstract] OR "growth hormone"[Title/Abstract] OR "somatotropin"[Title/Abstract])) AND ("Intervention study"[Title/Abstract] OR "controlled trial"[Title/Abstract] OR "randomized"[Title/Abstract] OR "randomized controlled trial"[Title/Abstract] OR "randomized clinical trial"[Title/Abstract] OR RCT[Title/Abstract])
