# Supplementary material for: Growth Hormone Response to L-Arginine Alone and Combined with Different Doses of Growth Hormone-Releasing Hormone: A Systematic Review and Meta-Analysis
Source: Int J Endocrinol. 2022 Nov 23;2022:8739289. doi: 10.1155/2022/8739289 (PMC9712012; doi:10.1155/2022/8739289)
Supplement: Supplementary Materials — Supplementary Table 1: GRADE evidence profile: Effect of Vitamin ARG and ARG + GRGH on GH. Supplementary Table 2: Study characteristics. Supplementary Table 3: Risk of bias assessment of the studies included in this meta-analysis. Appendix 1: PRISMA 2020 Checklist. Appendix 2: Search terms for Medline (PubMed). [file 8739289.f1.zip › Supplementary Table 1 (1).docx]

**Supplementary Table 1: GRADE evidence profile: Effect of Vitamin ARG and ARG+GRGH on GH**

|  | | **Certainty assessment** | | | | | | | | **No of Patients** | **Effect** | **Quality** |
| --- | --- | --- | --- | --- | --- | --- | --- | --- | --- | --- | --- | --- |
| **Outcomes** | **intervention** | | **No of studies** | **Study Design** | **Risk of bias** | **Inconsistency of results** | **Indirectness of evidence** | **Imprecision** | **Publication bias** |  | **Relative**  **(95% CI)** |  |
| GH | ARG | | 9 | RCT | not serious | serious | not serious | not serious | serious | 258 | M.D=10.07 (7.87, 12.28) | **Moderate** |
| GH | ARG+GRGH | | 5 | RCT | not serious | serious | not serious | not serious | serious | 464 | MD= 24.96 ( 17.51, 32.42) | **Moderate** |

ARG: Arginine, GH: growth hormone, GHRH: GH releasing hormone
