# Supplementary material for: Growth Hormone Response to L-Arginine Alone and Combined with Different Doses of Growth Hormone-Releasing Hormone: A Systematic Review and Meta-Analysis
Source: Int J Endocrinol. 2022 Nov 23;2022:8739289. doi: 10.1155/2022/8739289 (PMC9712012; doi:10.1155/2022/8739289)
Supplement: Supplementary Materials — Supplementary Table 1: GRADE evidence profile: Effect of Vitamin ARG and ARG + GRGH on GH. Supplementary Table 2: Study characteristics. Supplementary Table 3: Risk of bias assessment of the studies included in this meta-analysis. Appendix 1: PRISMA 2020 Checklist. Appendix 2: Search terms for Medline (PubMed). [file 8739289.f1.zip › Supplementary Table 2 (1).docx]

| **Supplementary Table 2:** study characteristics | | | | | | | | | | | | | |
| --- | --- | --- | --- | --- | --- | --- | --- | --- | --- | --- | --- | --- | --- |
| Author | Year | Country | Type of study | Sample size | sample | Age  (year) | health status | other substance were combined with ARG | ARG type | Dose of intervention substance | type of usage | outcome | results |
| Castagno,M | 2018 | Italy | cohort | 369  (224 male,  145 female) | blood | 2 to18 | healthy short structure(height less than 3rd percentile | GHRH | hydrochloride | ARG:0.5gr/kgiv, GHRH: 1 g/kg iv | iv | GH was measured in 0, 30, 45, 60 min | GH T0′ values, in the whole population, were significantly lower in males than in females and in pubertal than in pre pubertal. The GH peak level was higher in pre-pubertal than  in pubertal subject, whereas no difference was found according to  gender. The peak was at 45min. |
| Rigmonti,A | 2017 | Italy | RCT | 24 (14 male, 10 female) | blood | 10.8 | Prader willy syndrome (group1 :idiopathic short status group), group2: GH deficiency) | - | hydrocholoride | GHRH 1 μg/kg as i.v and 0.5 g/kg (maximum dose 30 g) of ARG hydrochloride | iv | GH was measured at−15, 0, 30, 45, 60, 90 and 120 minutes. | The ratios of circulating levels of 22 kDa- to 20 kDa-GH remained constant after GHRH plus ARG both in obese/non-obese and GHD/non-GHD groups, thus suggesting the preservation of a normal balance in GH isoforms in PWS. |
| Deutschbein,T | 2016 | Germany | RCT | 87 patients growth hormone deficiency:51(female:21 male:30) growth hormone suficiency:36(female15 male21) | blood | GHD(females 49.6 male49.9)GHS(female51 male44.4) | hypothalamic pituitary disease |  |  | GHRH (1 μg/kg) by an i.v. bolus, L-arginine (30 g)iv | iv | GH was measured at 0, 30, 45, 60, 90, and 120 minutes. | GH peak is 6.5 vs. 9.7 ng/ml in lean, 3.5 vs. 8.5 ng/ml in overweight, and 2.2 vs. 4.4 ng/ml in obese. BMI and sex account for most of the variability of peak GH levels |
| Khodary,EL | 2016 | USA | RCT | control(5 male and 6 female)  obese (7male and 5female) | blood | Control (21-33)  cases(17-23) | obese individual |  |  | arginine 5.0 ng/ml | iv | GH was measured at 0,30,60,90,120,150 and 180 minute. | There was a significant increase in plasma growth hormone response to arginine following weight loss in both male and female subjects, but the growth hormone response of the partially thinned obese female subjects remained significantly less than that of the normal volunteers. |
| Rigamonti, A | 2015 | Italy | RCT | 14(5male,9female) | blood | 19 | prader willy syndrome |  | hydrocholoride | GHRH1 μg/kg as i.v.and Argenine 0.5g/kg iv | iv | The peak of GH was measured. | The same ratios of GH isoforms in young PWS patients, suggesting that the hypothalamic dysfunction in this genetic disorder does not alter the qualitative and quantitative composition of GH isoforms present in circulation |
| Marostica,E | 2013 | Italy | RCT | group1:29children(21male,8female),group2:65adult(24malem41female) | blood | 18–41.2 | praderwili syndrome |  | hydrochloride | GHRH: 1 μg/kg as i.v. bolus at 0 minutes) and ARG: 0.5 g/kg (maximum dose 30 g) | iv | GH was measured at −15, 0, 30, 45, 60, 90 and 120 minute. | The quantitative and qualitative analyses of GH responsiveness to GHRH + arginine highlight relevant differences between PWS children and PWS adults and genotype-related traits. |
| Zajac.A | 2010 | Poland | cade-control | 9control,8intervention | bood | control:22.8+1.7,interventioon:23.5+2.1 | normal athletes | ornithine | hydrochloride | ARG:3gr,ornithine:2.2gr | oral | GH was measured 2 min and 1 hour after ingestion | Average GH level was significantly increased in both investigated groups at the end of the last exercise trial and remained significantly above the baseline 1 hour later. The group that received the arginine and orn supplementation demonstrated significantly higher GH levels at both time points |
| Corneli,G | 2007 | Italy | RCT | 152 lean patients (85 males and 67 females) subgroup1(panhypopituitary):35  subgroup2(1or2pitutary disease):18  subgroup3(nopitutarydisease):99,control:201 patients | blood | 19.2 | structural hypothalamic pituitary abnormalities | GHRH | hydrocholoride | argenin :0.5 g/kg,GHRH:1 mg/kg | iv | Peak GH responses | No age or gender differences were found between controls and patients The mean peak GH in control subjects (69.3G 2.6 mg/l) was significantly higher than that in patients |
| Keller,A | 2007 | Germany | Clinical terial | 30 patients | blood | 9 | GHD |  | hydrochloride | ARG:0.5g/kg max 30gr | iv | GH was measured at -30, 0,  15, 30, 45, 60, 90 and 120 min. | Arg + GHRH gave higher GH levels than insulin or Arg alone. |
| Collier,S.R | 2006 | USA | RCT | 8 | blood | 18-25 | young male, healthy, with no major chronic diseases such as diabetes, cardiovascular disease, atherosclerosis, hypertension, or dyslipidemia | non | not mentioned | ARG:7gr | oral | 22KD, 20KD GH was measured in plasma. | Peak blood GH concentrations were found 60 min after the arginine ingestion on both the Ex and ArgEx day and remained elevated until the end of exercise, at which point the blood concentrations decayed toward resting concentrations. The peak was greater in exercise group than ARG+exercise, the ARG alone had the lower level of GH. |
| Grugni,G | 2006 | Italy | RCT | 44 praderwili syndrome,17 obese control | blood | 18–41 | praderwili syndrome |  | hydrochloride | GHRH (1 μg/kg) by an i.v. bolus, L-arginine (30 g)iv | iv | GH was measured at −15 and 0 minutes and then 30, 45, 60, 90 and 120 minutes. | The GH response to GHRH + ARG was significantly lower in PWS patients |
| Collier,S.R | 2005 | USA | RCT | 8 | blood | 24.8 | healthy young males | non | not mentioned | ARG:5, 9 or 13 g of arginine oral | oral | 22KD, 20KD GH was measured in plasma. | Resting GH concentrations on each study day were not different between the placebo, 5, 9 and 13 g day. Most subjects showed an increased GH response with arginine ingestion, ex cept on the 13 g day, as high doses of amino acids can cause GI distress due to the osmotic movement of water into the stomach and intestine. |
| Corneli,G | 2005 | Italy | RCT | 318 control:(147 men and 171 women322), intervention (174 men and 148 women) | blood | group1:47.6,group2:48.0,control:39.9 | organic hypothalamic-pituitary disease | GHRH | hydrocholoride | argenin :0.5 g/kg,GHRH:1 mg/kg | iv | Peak GH responses | The peak always occurred between 30 and 60 min. Peak GH responses were significantly higher in lean than in overweight or obese. Responses to the GHRH-ARG test and BMI was found in PHD patients without GHD but not in those with GHD. |
| Maghnie,M | 2002 | Italy |  | 36 patients acquired, group1: GH deficiency including idiopathic pituitary stalk thickness (n 15), group2: LCH affecting the hypothalamic-pituitary region (n 11), and group3: craniopharyngioma (n 10). | blood | children:8.5,adults:26 | prader-wili patients |  | hydrochloride | GHRH: 1 μg/kg as i.v. bolus at 0 minutes) and ARG: 0.5 g/kg (maximum dose 30 g) | iv | GH was measured at 0, 15, 30, 45, 60, 75, and 90 min | The patients GHRH-plus-ARG test stimulates GH response to a so-called: normal value, suggesting that pituitary responsiveness to GHRH plus ARG may fail to recognize acquired GHD. |
| Aimretti,G | 2000 | Italy | RCT | normal subjects: 48 young,patients:62 GHD:18,GH neurosecretory dysfunction: 21,organic hypopituitarism with GHD: 18 | blood | 26-27 | growth hormone deficiency |  | hydrochloride | GHRH (1 μg/kg) by an i.v. bolus,ARG (ARG hydrochloride, 0.5 g/kg iv | iv | Peak of GH was measured. | The mean GH peak after GHRH1ARG was higher than that after ITT. In conclusion, given appropriate cut-off limits, GHRH/ARG is as reliable as ITT for retesting patients who had undergone GH treatment in childhood. |
| Marcell,T | 1999 | USA | RCT | 20young(9female,11male),8old(3female,5male) | blood | group1: 47.1,group2: 46.0 | non-insulin-dependent diabetes mellitus (NIDDM) |  | hydrochloride | GHRH: 1 μg/kg as i.v. bolus at 0 minutes) and ARG: 0.5 g/kg (maximum dose 30 g) | oral | GH was measured at 10, 20, 40, and 60 minutes. | Oral Arg alone did not result in any increase GH secretion at rest. When Arg was coadministered during exercise, GH release was not affected in either the young or old and appeared to be blunted in the young compared to the exercise onlytrialin the young. |
| Maccario,M | 1996 | Italy | RCT | group1:(Seven female patients with idiopathic hyperprolactinemia(N = 3) or tumoral ( = 4, microprolactinomas)) and group2:(seven female with abdominal obesity)group3:7normal female | blood | young:22.1,old:68.5 | hyperprolactinemic patients |  | hydrovhrolide | ARG:0.5g/kg | iv | GH was measured at 0,30,60,90 and 120 minutes. | GH levels decreased after glucose and increased after ARG administration. This latter response was inhibited significantly by previous glucose adminis¬ tration in normal subjects and hyperprolactinemia. In obese the GH response to ARG was lower than in normal subjects and hyperprolactinemia. |
| Procopio,M | 1995 | Italy | RCT | seven obese and ten healthy | blood | 19 | obese individual |  | hydrocholoride | GHRH1 μg/kg as i.v.and Argenine 0.5g/kg iv | iv | GH was measured at -15,0,30,60 and 90 minutes. | The GHRH-induced GH rise in obese was lower than in control. |
| Martina,V | 1995 | Italy | RCT | 14 patients with non-insulin-dependent diabetes mellitus (NIDDM)(group1:7normal weight,group2:7obese),12 control: group1(12 obese patients(7male,5female)and group2:12 healthy(6male,6female) | blood | 38.4 | non-insulin-dependent diabetes mellitus (NIDDM) |  | hydrochloride | GHRH: 1 μg/kg as i.v. bolus at 0 minutes) and ARG: 0.5 g/kg (maximum dose 30 g) | iv | GH was measured at-15,0,30,45,60,75 and 90 minutes. | r results demonstrate that, irrespective of body weight, patients with non insulin-dependent diabetes mellitus have an impairment of GH response to GHRH alone or com bined with arginine. This finding may be explained by an in hibition of high FFA levels on GH secretion or alternatively by an inhibition of chronic hyperglycemia. |
| Ghigo,E | 1994 | Italy | RCT | 27 normal elderly(11 male 16 female) GROUP1(7)GROUP2(6)GROUP3(7)group4(7) | blood | 35- 2.1 | Anorexia nervosa |  | hydrochloride | GHRH (1 μg/kg) by an i.v. bolus, L-arginine (30 g)iv | iv | GH was measured at -60 and 0 min and then every  15 min until +90 min. | The GH response to ARG + GHRH was  similar to Anorexia nervosa patients and in normal individuals. |
| Ghigo,E | 1994 | Italy | RCT | cases:25.8,control:28.0 | blood | 70-86 | normal elderly individuals |  | hydrochloride | GHRH (1 μg/kg) by an i.v. bolus, L-arginine iv, group1(30gr)iv,group2(10gr)iv,group3(5gr)iv,group4(8gr)oral | oral | GH was measured before and after usage. | no difference was present in the GH response to  GHRH alone between male and females or amongst  groups. the GH response  to GHRH in elderly subjects is enhanced even by  low iv doses of arginine and by the orally |
| Bauman,W.A | 1994 | USA | RCT | 16 control-16 intervention | blood | control:39 ,intervention: 45 | spinal cord injery | non | hydrochloride | ARG:30gr | iv | GH was measured in -30 , 0, 30, 60 90 and 120 minutes | Plasma hGH values at 30 and 60 minutes were significantly lower in the group of patients, also the peak is lower in this group. |
| Bellone,J | 1993 | Italy | RCT | 31 children(11 boys and 20 girls, pubertal stage I-III | blood | 5.5-13.8 | normal children with familial short stature | non | aspartat and hydrocholoride | ARG:4gr oral,0.5gr/kgiv, GHRH: 1 f.!g/kg iv | oral and iv | GH was measured in-60, -30, 0, 15, 30, 45,  60, 75, 90 min. | The GH response to iv administered ARG-H did not  significantly differ from that observed after oral  ARG-H but was significantly higher (p<0.05) than  that after oral ARG-A. The GH responses to GHRH  alone or combined with iv or oral ARG were similar  in all groups. |
| Hanew,T | 1993 | Sendai | RCT | 90patients with idiopatic GH deficiency(11male,8female),3patients with secondry GHD(male),7short but normal children(5male,,2female) | blood | 29-32 | idiopatic GH deficiency, |  | hydrochloride | 100microgr of synthetic GHRH,ARG:0.5 g/kg i.v. over 30 min) | iv | GH was measured at -30,0,60 and 120 minute after ingection. | These results indicate that hypothalamic lesions were primarily  responsible for GH deficiency in about 60% of the patients with idiopathic growth hormone deficiency. |
| Corpas,E | 1992 | USA | RCT | 8 control,8intervention | blood | 22-33 | healthy | Lysine | hydrocholoride | ARG:1.5 plus 1.5 g lysine | oral | GH was measured at15, 30,45, 60, 90, and 120 minutes | GH responses were similar, in old vs young men. Arginine/lysine did not significantly alter spontaneous or GHRH-stimulated GH levels. Increases in serum GH after a single dose of arginine/lysine was similar in old and young groups |
| Ghigo,E | 1992 | Italy | RCT | 7 normal | blood | Eight obese subjects (two males and six females)eight normal volunteers (five men and three women) | obese individual |  | hydrochloride | GHRH (1 μg/kg) by an i.v. bolus, L-arginine (30 g)iv | iv | GH was measured at-45, -30. -15.0, 1.5, 30.45,60, 75. and 90 minutes. | Mean basal serum GH levels were similar in all tests. The  administration of GHRH induced a clear-cut increase in  GH values. |
| Ghigo,E | 1992 | Italy | RCT | 11 women Anorexia nervosa 20 control | blood | cases:18.8 control:22.0 | obese individual |  | hydrochloride | GHRH (1 μg/kg) by an i.v. bolus, L-arginine (30 g)iv | iv | GH was measured at - 15 and 0 minutes, and then every 15 minutes until 90 minutes. | Basal serum GH levels were similar in obese and normal  subjects. In the obese patients GHRH administration induced a GH response that was  significantly lower. |
| Soliman,A.T | 1987 | Egypt | cade-control | 8 control-10 intervention | bood | control: 11.2, intervention : 13 | children with vitamin D deficiency rickets | non | hydrochloride | ARG:0.5g/kg | iv | GH was measered in30,60,75 and 90 min. | there was no significant difference between control and rachitic children in responsive to ARG and ornithine. |
| Besset,A | 1982 | France | RCT | 5 | blood | 21-35 | healthy male | non | aspartat | ARG:250 mg/kg/day | oral | GH was measured in day and at night. | Following chronic arginine aspartate administration the major peak associated with early sleep was increased. a major increase of about 58% was noticed in the mean night-time secretion. The day-time secretion was not midified due to ARG. |
| Sizonenk,P.C | 1975 | Switzerland | RCT | group1:normal children(10 children and adolescents (4 females and 6 males)),group2(idiopatic short stature:18 subjects (2 females and 16 males)),group3:(consisted of 6 subjects (1 female and 5 males) with isolated GH deficiency) | blood | 10.8 | idiopatic short stature children |  | monochloride | ARG:20 g/m2 | iv | GH was measured at-30,0,30,60,90 and 120 minutes. | Arginine infusion resulted in two- to threefold increases of plasma IRG in the normal group, and similar increases were observed in all of the other groups tested. |
| Kalk,W.J | 1974 | South Africa | RCT | over all 21 pancreatitis patients(13male,8female)(4 different groups(diabetic n=10,immunoreactive insulin responders n=10,Insulinopenic patients n==11,normal GTT n=11) | blood | 21-64 | pancreatitis patients |  | monohydrocholoride | ARG:30 g | iv | GH was measured at 10, 20, 30, 40, 50, 60, 75 and 90minutes. | Basal I~GH  levels and peak responses to arginine were normal but  were uninfluenced by sex, glucose intolerance, fasting  hyperglycaemia or insulinopenia. |
| Gacs,C | 1973 | Hungary | RCT | 15 control 13cases(corticosteroid treatment patients) | blood | cases:9.9 control:8.7 | Children with nephrosis had no proteinuria or hypoproteinemia |  |  | ARG:0.5 gm/kg/body | iv | GH was measured at 0, 30, 60 and 90 minutes | The growth hormone level was higher in the steroid group than in the controls at every point of time. The difference was significant at 60 and 90 minutes. |
| Koncz,L | 1973 | USA | RCT | 15women patients(group1(>8 years diabetes):8,group2(<8years diabetes):7),7normal(group1: | blood | group1:18-46,group2:23-52,group3:26-32 | juvenile-type diabetes |  | monohydrochloride | ARG:0.35 ± 0.01 gm. per kilogram | iv | GH was measured at -40,-20,0,20,40 and 60 minutes . | Diabetics with chronically elevated blood glucose levels had a blunted growth hormone response to arginine. In patients with diabetic retinopathy, growth hormone response was somewhat blunted, probably due to higher blood glucose level. |
| Root,A.W | 1969 | USA | RCT | control25 children (10 girls and 15 boys)-intervention70 children (7 girls and 10 boys) | blood | intervention:5-17,control:1-16 | anteriorhypopituitarism or hypothyroidism | non | hydrochloride | ARG:0.5 mg. per kilogram | iv | GH was measured in -30, 0, 15, 30, 45, 60, 90, and 120 minutes. | peak response was at60 min the peak was:(female, 16.3 _+4.4; male, 14 + 3.1 m/zg per milliliter) . there was not any increase in 4 subjects. The GH concentration was low in controls. |
| Merime,T.J | 1969 | USA | RCT | 8 normal | blood | 20 -28 | healthy women in different menstural period | non | hydrochloride | ARG:1/2-1/6 gr per poud of body weight. | iv | GH was measured in 0,30,60,90 min of mid-cycle and in menstrual duration | peak was accrued in 60 min. The mean increment of plasma HGH at midcycle  was likewise greater than during active menstrual period. |
| Copinchi,G | 1967 | USA | RCT | 10 control(5men and5 female), 10 intervention (5men and 5female) | blood | 21-54 | obese individual | non | monohydrocholoride | ARG:30 gm | iv | GH was measured every 30 min to 120 min. | Higher in the normals than in the obese subjects. the peak was at 60min. |
| Knopf,F.J | 1965 | USA | RCT | 6healthy female, 3 hypopituitarism | blood | 9 | hypopituitarism female |  | monohydrochloride | 30 g. arginine | iv | GH was measured at 0,30,60,90,120 minutes. | The growth hormone response was absent in 3 hypopituitary patients and was decreased in a single obese person. Arginine may have advantages as a provocative test for the adequacy of H.G.H. output |
| PHD: pituitary hormone deficient, GH :growth hormone, GHD :growth hormone deficiency, RCT : Randomized clinical trial | | | | | | | | | | | | | |
