# Supplementary material for: Growth Hormone Response to L-Arginine Alone and Combined with Different Doses of Growth Hormone-Releasing Hormone: A Systematic Review and Meta-Analysis
Source: Int J Endocrinol. 2022 Nov 23;2022:8739289. doi: 10.1155/2022/8739289 (PMC9712012; doi:10.1155/2022/8739289)
Supplement: Supplementary Materials — Supplementary Table 1: GRADE evidence profile: Effect of Vitamin ARG and ARG + GRGH on GH. Supplementary Table 2: Study characteristics. Supplementary Table 3: Risk of bias assessment of the studies included in this meta-analysis. Appendix 1: PRISMA 2020 Checklist. Appendix 2: Search terms for Medline (PubMed). [file 8739289.f1.zip › Supplementary Table 3 (1).docx]

| **Supplementary Table 3: Risk of bias assessment of the studies included in this meta-analysis** | | | | | | | | |
| --- | --- | --- | --- | --- | --- | --- | --- | --- |
| Author | year | Random Sequence Generation | Allocation concealment | Selective outcome reporting | Other sources of bias | Blinding of participants personnel | Blinding of outcome assessors | Incomplete outcome data |
| Floyd, J. C., | 1965 | L | H | H | L | H | H | L |
| CEORGES COPINSCII | 1967 | L | L | H | L | H | H | L |
| thomas J.Meriemee | 1967 | L | L | H | L | L | H | L |
| Allen W. Root | 1969 | L | L | H | L | L | H | L |
| THOMAS J. | 1969 | L | U | H | H | L | H | L |
| G. Gacs | 1973 | L |  | L | H | L | H | L |
| Lajos Koncz | 1973 | L | L | H | L | H | H | L |
| W.J. Kalk, | 1974 | L | L | H | H | L | H | L |
| PIERRE C. SIZONENK0,' | 1975 | L | L | L | H | L | H | L |
| A. Besset | 1982 | L | H | H | H | L | H | L |
| Ashraf T | 1987 | L | H | H | H | H | H | L |
| E. Ghigo | 1992 | L | H | H | L | L | H | L |
| E. Ghigo | 1992 | L | U | L | L | H | H | L |
| W. A. Bauman | 1993 | L | L | U | H | H | H | L |
| J. Bellone | 1993 | L | H | H | L | H | H | L |
| Emiliano Corpas | 1993 | L | L | L | L | H | H | L |
| Tohoku J. Exp. Med., | 1993 | L | L | L | H | L | H | L |
| S. Loche | 1993 | L | L | L | L | H | H | L |
| E. ARVAT, | 1994 | L | U | H | H | H | H | L |
| E. Ghigo | 1994 | L | L | H | H | H | H | L |
| E. Ghigo | 1994 | L | L | H | L | U | H | L |
| V. Martina | 1994 | L | L | H | L | H | H | L |
| M. Procopio | 1995 | L | H | L | H | L | H | L |
| Mauro Maccario | 1996 | L | H | L | L | L | H | L |
| Allen W. Root | 1996 | U | L | H | H | H | H | L |
| TaylorJ. Marcell | 1999 | H | L | H | H | H | H | L |
| G. AIMARETTI | 2000 | L | L | H | H | L | H | L |
| MOHAMAD MAGHNIE | 2002 | L | L | H | L | L | H | L |
| Scott R | 2005 | L | H | L | H | H | H | L |
| Ginevra Corneli | 2005 | L | L | H | H | L | H | L |
| S. R. Collier | 2006 | L | H | H | H | H | H | L |
| G. Grugni | 2006 | L | U | L | L | H | H | L |
| Ginevra Corneli | 2007 | L | L | H | H | L | H | L |
| A. Keller1, | 2007 | L | U | H | H | L | H | L |
| ADAM ZAJAC | 2010 | L | H | H | L | L | H | L |
| Anneke J. A. H. van Vught | 2012 | L | L | H | H | L | H | L |
| E. Marostica | 2013 | U | L | L | H | L | H | L |
| A.E. Rigamonti | 2015 | L | L | H | H | H | H | L |
| Timo Deutschbein | 2016 | L | L | H | H | L | H | L |
| ASHRAF Z. EL-KHODARY, | 2016 | L | L | H | H | L | H | L |
| Antonello E | 2017 | L | L | L | H | L | H | L |
| M. Castagno | 2018 | L | L | H | H | H | H | L |
| H: high risk, L: low risk, U: unclear risk | | | | | | | | |
